# ABCA1 is an extracellular phospholipid translocase

**DOI:** 10.1038/s41467-022-32437-3

**Published:** 2022-08-16

**Authors:** Jere P. Segrest, Chongren Tang, Hyun D. Song, Martin K. Jones, W. Sean Davidson, Stephen G. Aller, Jay W. Heinecke

**Affiliations:** 1 Department of Medicine, Vanderbilt University Medical Center, Nashville, TN USA; 2 Department of Medicine, University of Washington, Seattle, WA USA; 3 Department of Pathology and Laboratory Medicine, University of Cincinnati, Cincinnati, OH USA; 4 Department of Pharmacology & Toxicology, University of Alabama at Birmingham, Birmingham, AL USA

**Keywords:** Computational biophysics, Molecular modelling, Permeation and transport, Membrane proteins

## Abstract

Production of high density lipoprotein (HDL) requires ATP-binding cassette transporter A1 (ABCA1) to drive phospholipid (PL) from the plasma membrane into extracellular apolipoprotein A-I. Here, we use simulations to show that domains of ABCA1 within the plasma membrane remove PL from the membrane’s outer leaflet. In our simulations, after the lipid diffuses into the interior of ABCA1’s outward-open cavity, PL extracted by the gateway passes through a ring-shaped domain, the annulus orifice, which forms the base of an elongated hydrophobic tunnel in the transporter’s extracellular domain. Engineered mutations in the gateway and annulus strongly inhibit lipid export by ABCA1 without affecting cell-surface expression levels. Our finding that ABCA1 extracts lipid from the outer face of the plasma membrane and forces it through its gateway and annulus into an elongated hydrophobic tunnel contrasts with the alternating access model, which proposes that ABCA1 flops PL substrate from the inner leaflet to the outer leaflet of the membrane. Consistent with our model, ABCA1 lacks the charged amino acid residues in the transmembrane domain found in the floppase members of the ABC transporter family.

## Abstract (web summary)

ATP-binding cassette transporter A1 (ABCA1) drives phospholipid (PL) from the plasma membrane into extracellular apolipoprotein A-I, for the production of high density lipoprotein (HDL). Here, the authors use simulations to assess the mechanism of ABCA1 function and show that ABCA1 extracts lipid from the outer face of the plasma membrane.

## Introduction

Unregulated accumulation of cholesterol from atherogenic lipoproteins is a central feature of atherosclerosis. In contrast, many lines of evidence suggest that high density lipoprotein (HDL) is cardioprotective, in part because it promotes cholesterol export from macrophages of the artery wall^1,2^. The underlying mechanism involves ABCA1, an ATP-binding cassette (ABC) transporter that uses energy derived from ATP to export phospholipid (PL) out of the plasma membrane.

The 49 members of the human ABC membrane transporter superfamily are classified into five subfamilies, A to G^3,4^. The subfamilies A, B, C, D, and G, including ABCA1, translocate substrates across the plasma membrane^5^. The A subfamily’s physiological importance is demonstrated by its association with a wide variety of inherited diseases, such as fatal surfactant deficiency (ABCA3)^6^, Stargardt disease (ABCA4)^7^, Harlequin ichthyosis (ABCA12)^8^, and schizophrenia and bipolar disorder (ABCA13)^9^. Mutations in ABCA1 cause Tangier disease, a rare genetic disorder that impairs cholesterol export from cells, resulting in cholesterol accumulation by tissue macrophages and very low levels of HDL cholesterol (HDL-C)^10,11^.

Membrane ABC transporters share a common architecture of two transmembrane domains (TMDs) and two nucleotide-binding domains (NBDs). ABC half-transporters contain a single TMD and a single NBD that form a hetero- or homo-dimeric structure to transport substrate. ABC full-transporters are a single polypeptide that contains two TMDs and two NBDs.

The molecular structures of the full-transporter ABCA1 and of the dimeric complex of the half-transporters ABCG5/ABCG8 (which play key roles in sterol secretion into the gut and bile) have been solved^12,13^. The nucleotide-free G5/G8 structure adopts an inward-facing transmembrane cavity similar to that of other nucleotide-free ABC exporters. In contrast, nucleotide-free ABCA1 exhibits an outward-open transmembrane cavity, raising the possibility that the two proteins transport substrates by different mechanisms.

Apolipoprotein A-I (APOA1), the major HDL protein, binds directly to ABCA1 in the plasma membrane as well as to PL-enriched domains it generates outside the cell^14^. This binding promotes the export of PL and cholesterol from cells, though the underlying mechanisms remain unclear. Phillips proposed that ABCA1 translocates PL from the inner to the outer leaflet of the plasma membrane, causing a bulge that provides an active surface where cholesterol accumulates and APOA1 is transformed into a nascent HDL particle^15^. Nagata et al.^16^ suggested that lipid efflux involves the conversion of ABCA1 monomers into homodimers. In turn, the homodimers bind two APOA1 molecules that are subsequently lipidated by ABCA1.

The generally accepted model for substrate export by ABC transporters, called the alternating-access mechanism, envisions a switch between an inward-facing transmembrane cavity and an outward-facing transmembrane cavity^17^. For ABCA1, the inward-open transmembrane cavity is proposed to accept PL from the inner leaflet of the plasma membrane^5^, which then closes when the NBDs bind ATP and vectorially transport the substrate into the extracellular space. In this model, PLs in the inner leaflet of the plasma membrane diffuse into the inward-open transmembrane cavity and then are translocated when they swing through 180° rotation to assume the orientation of outer leaflet lipids. However, such a reorientation would be energetically costly, and it is unclear how inner leaflet PLs could undergo such a rotation^5^. Moreover, in the cryo-electron microscopy (cryo-EM) structure of ATP-free ABCA1^13^, the TMDs do not form an inward-facing transmembrane cavity, which would be required for the alternating-access mechanism^5,17^.

Here, we use simulations to show that ABCA1 extracts lipid from the outer face of the plasma membrane and forces it through its gateway and annulus into an elongated hydrophobic tunnel. Our model contrasts with the widely accepted alternating-access model, which flops PL substrate from the inner leaflet to the outer leaflet of the membrane.

## Results

**Fig. 1:**
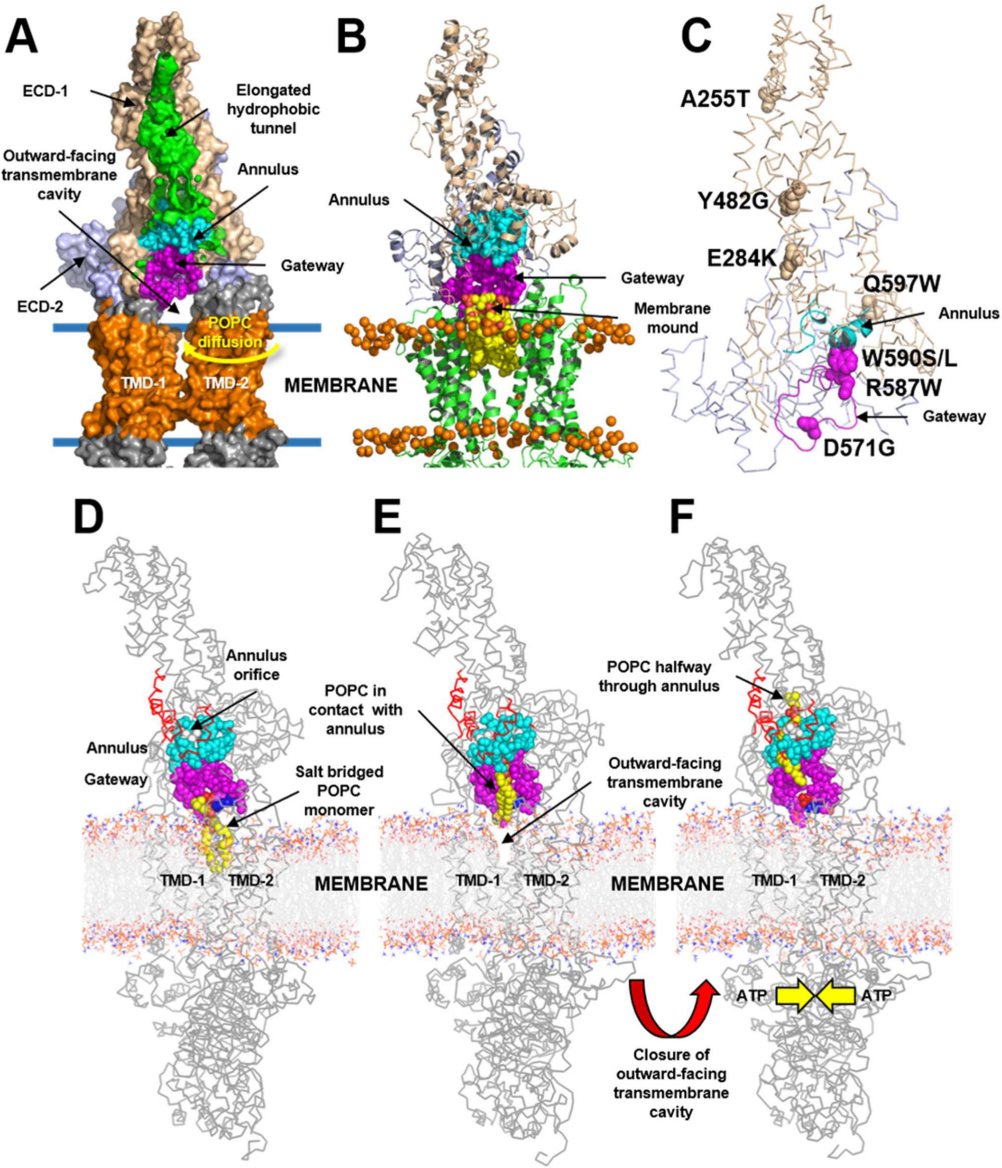
Details of POPC translocation from the outward-facing transmembrane cavity of ABCA1 through the gateway/annulus complex into the elongated hydrophobic cavity. The outward-facing transmembrane cavity, TMD-1 and TMD-2 (orange), ECD-1 (peach), and ECD-2 (light blue) and the elongated hydrophobic tunnel (green) were identified in the structure of ABCA1^13^. The elongated hydrophobic tunnel in ECD-1 was demarcated using PyMol cavity algorithm. **A** Gateway (residues 564–592, magenta) and annulus (residues 69, 71–80, 363, 368–379, cyan)*.* The annulus forms the bottom of the elongated hydrophobic tunnel. Shown is the pathway for diffusion of POPC from the outer leaflet of the membrane bilayer (blue lines) into the outward-facing transmembrane cavity (yellow arrow). **B** Final frame after 2 µs simulation of the ECDs and TMDs in a POPC bilayer (all-atom model). Five POPC molecules (termed the membrane mound, yellow space-filling) diffused from the outer membrane leaflet into the outward-facing transmembrane cavity (yellow arrow, **A**) and then displaced ~10–12 Å above the plane of the bilayer into the outward-facing transmembrane cavity. The gateway is the 29-residue charged loop of ECD-1 that binds the membrane mound in CGMD simulations. This loop is contiguous with the outward-facing transmembrane cavity on one side and the annulus of the elongated hydrophobic tunnel. **C** Location in the gateway of four of the eight Tangier disease point mutations identified in the ECD-1 of human ABCA1. **D–F** SMD Freeze-frames of the translocation of a single POPC molecule up to and through the annulus and partway into the elongated hydrophobic tunnel. The gateway and annulus are colored magenta and cyan, respectively. **D** The 1.9 μsec all-atom frame from the 10 μsec CGMD simulation of ABCA1 embedded in a POPC bilayer was the starting structure*.* The annulus orifice (residues 73-75, 77, 78, 371, 375) is shown in white. **E** The POPC was translocated by SMD to the annulus orifice. **F** The POPC then was translocated halfway through the annulus orifice. This process required significant energy in the SMD (Supplementary Fig. 4). To be energetically favorable in vivo, we propose that the outward-facing transmembrane cavity would close, likely from an ATP-dependent process, forcing the PL through a modified orifice.

To investigate the mechanisms that enable ABCA1 to export PL, we inserted the cryo-EM structure of ABCA1^13^ (wild type and mutated forms) into a 1-palmitoyl-2-oleoyl-sn-glycero-3-phosphocholine (POPC) membrane bilayer. Note the key structures of wild-type ABCA1: the outward-open transmembrane cavity, extracellular domains (ECDs), TMDs, and an elongated hydrophobic tunnel^13^ (Fig. 1A). We then used coarse-grained molecular dynamics (CGMD) to study interactions between PL and protein. This approach allowed us to monitor both the movements of individual PL molecules and changes in the structure of ABCA1 during CGMD simulations of wild type and two mutated ABCA1 structures.

### POPC molecules extracted from the outer leaflet of the membrane diffuse through the opening in the outward-open transmembrane cavity of ABAC1

We performed three 10 µs replica CGMD simulations of wild-type ABCA1 structures inserted into a POPC bilayer. The final frames after each 2 μs simulation of the three replicas, converted to all-atom models, demonstrated that 3–5 POPC molecules extracted from the outer leaflet of the membrane had diffused through the opening in the outward-open transmembrane cavity of ABCA1—between TMD-1 and TMD-2—and into the interior (shown schematically in Fig. 1A). After each 2 µsec simulation, the extracted POPC molecules had assembled into a complex, termed the membrane mound, ~10 Å above the plane of the lipid bilayer (Fig. 1B). At the end of each of the three 10 µs replica CGMD simulations, the mound had collapsed into a single POPC molecule, indicating that the mound was transient.

**Fig. 2:**
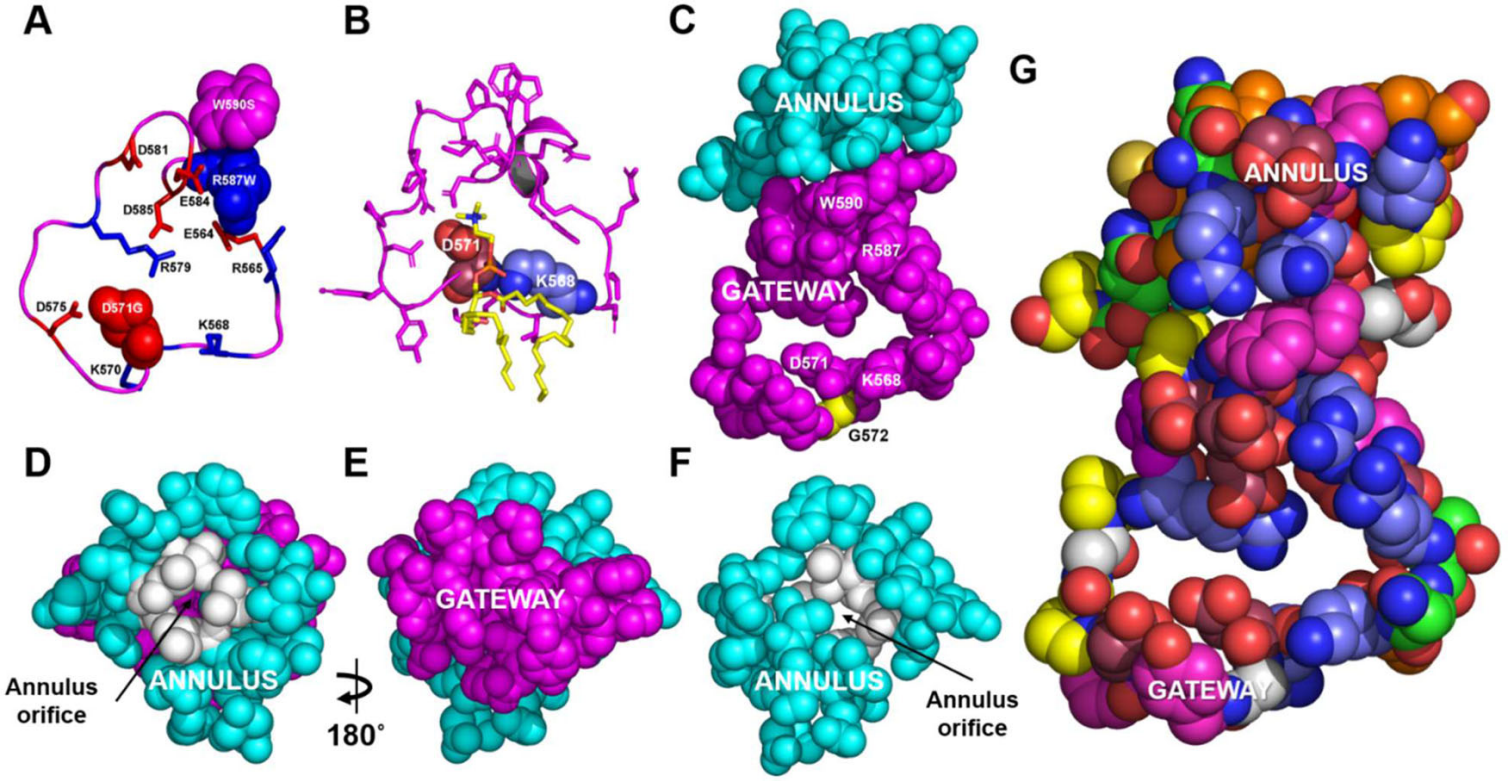
Structural details of the gateway/annulus complex. **A**, **B** The backbone of the gateway (magenta). **A** The basic (blue, stick) and acidic (red, stick) amino acid residues of the gateway (residues 564–592, magenta) from the ABCA1 structure determined by cryo-EM^13^. The three amino acid residues in the gateway known to be mutated in Tangier disease (four point mutations in total) are shown in the space-filling mode. **B** The POPC molecule (yellow stick) forms salt-bridges to residues D571 and K568 (space-filling red and blue, respectively) in the 1.9 µsec frame during coarse-grained molecular dynamics (CGMD) modeling of unmutated ABCA1. All side chains but residues D571 and K568 are magenta stick. **C** The gateway (magenta)/annulus (cyan) complex. Residues of the elongated hydrophobic tunnel that lie within 10 Å of any gateway residue form the annulus domain (residues 69, 71–80, 363, and 368–379). **D** The annulus (cyan) viewed from the side opposite the outward-facing transmembrane cavity*.* Note the small orifice (residues 73-75, 77, 78, 371, 375, colored white) in the middle of the annulus through which magenta-colored residues of the gateway on the opposite side are visible. **E** The base of the annulus (D) rotated 180° around the y-axis to show the gateway. The view is from the outward-facing transmembrane cavity. **F** Representation of the annulus with the gateway removed to display the annulus orifice. **G** The amino acid composition of the gateway/annulus complex. Acidic residues, rose; basic residues, blue; aromatic residues, magenta; hydrophobic residues, orange; prolines, yellow; neutral residues, green; glycines, white.

Inspection of the cryo-EM structure revealed a loop of ABCA1 (residues 564–592) at the base of the elongated hydrophobic tunnel (Figs. 1A and 2A). This loop was contiguous with the outward-open transmembrane cavity on one side and the transverse hydrophobic tunnel on the other^13^. The large open loop contained 29 amino acid residues of which 5 were basic, 6 were acidic, 5 were aromatic and 7 were hydrophobic. Several of these charged residues were in close contact with the head groups of the POPCs that had diffused into the interior of the protein to form the membrane mound (Fig. 1B).

### Extracted POPC molecules diffuse through the gateway toward the elongated hydrophobic tunnel of ABCA1

**Fig. 3:**
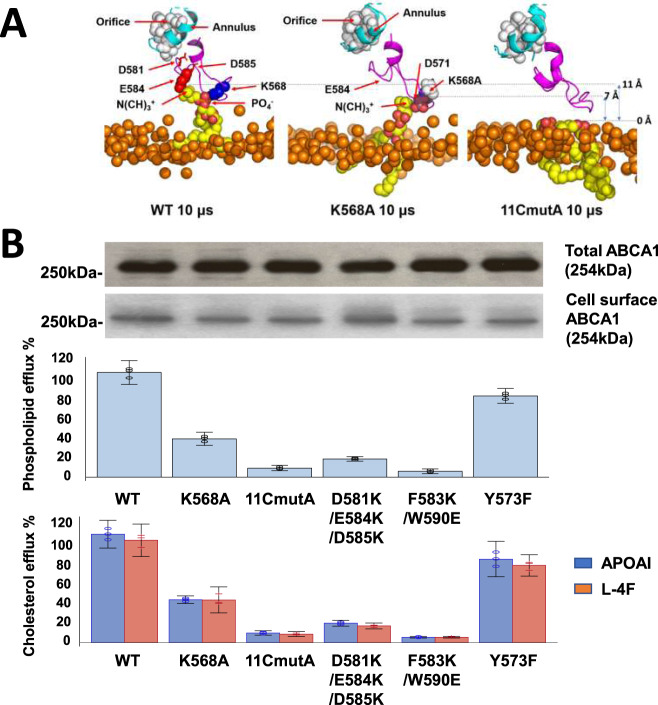
Charged amino acids in the gateway promote PL extraction and lipid export by ABCA1. **A** Extraction of POPC molecules from the membrane bilayer into the gateway by wild-type and mutated ABCA1. Final frames of the three 10 μsec CGMD simulations showing the gateway (magenta, cartoon), annulus (cyan, cartoon), and annulus orifice (white, space-filling) for different ABCA1 monomers inserted into a POPC bilayer. The ABCA1 monomers are wild-type (WT, left panel), K568A (middle panel), and 11CmutA (mutation of all 11 charged residues to alanine, right panel), respectively. The position of the upper monolayer surface in each image is shown by orange space-filling phosphorus atoms. The single POPC molecule extracted by the ABCA1 monomer of WT and K568A are shown in yellow (space-filling). The 11CmutA monomer failed to extract POPC (yellow, space-filling). Left panel, K568 is space-filling blue, E584 is space-filling red and D581 and D585 are stick red; middle panel, K568A is space-filling white, D571 is space-filling red. **B** Effects of gateway mutations K568 and 11CmutA on PL and cholesterol efflux by ABCA1. Wild-type, K568A, 11CmutA, D581K/E584K/D585K, F583K/W590E, and Y573F ABCA1 were expressed in BHK cells. PL and cholesterol efflux by the cells were quantified after incubation with APOA1 or an equal weight of L-4F, an APOA1-peptide mimetic. Lipid efflux and ABCA1 expression were quantified as described in Methods. Relative cholesterol and phospholipid efflux are normalized to WT and are presented as mean ± SD of three independent experiments with three replicates per experiment. Source data are provided as a Source Data file.

We term the loop formed by the residues the gateway for three reasons. First, POPC molecules from the membrane diffused partway into the loop toward the elongated hydrophobic tunnel of ABCA1. Second, four point mutations that cause Tangier disease (two shown not to affect cell-surface expression of ABCA1) are located in the loop (D571G, R587W, W590S/L) (Fig. 1C). Third, mutating positively and negatively charged amino acid side chains in the loop blocked the movement of membrane PL into the interior of ABCA1 in our simulations. They also inhibited PL and cholesterol export by ABCA1 from cultured cells (Fig. 3B).

### The annulus regulates the movement of POPC into the elongated hydrophobic tunnel

The position of the elongated hydrophobic tunnel relative to the gateway is shown in Fig. 1A. A structure, termed the annulus (residues 69, 71–80, 363, 368–379), forms a compact domain at the tunnel’s base (Fig. 1A). By definition, this domain contains all residues at the base of the elongated hydrophobic tunnel within 10 Å of gateway residues (Figs. 1D and 2C). By mutating key residues, we determined that the central portion of this domain, termed the annulus orifice (Fig. 2D; residues 73–74, 77, 371, 375), helps regulate PL export by ABCA1.

To test if the charged residues of the gateway are important in formation of the lipid mound, we analyzed the 3 replicas of the 10 µs CGMD simulation of wild-type ABCA1 to quantify how often the nearest four charged residues of the gateway (K568, K570, D571, and D575) form salt bridges with POPC in the membrane mound. K568 formed salt bridges with the PO_4_^-^ group of POPC 77% of the time, about twice as often as D571; D571 formed salt bridges with the choline group 39% of the time, 1.6 times as often as D575 (Supplementary Table 1). These observations suggest that K568 and to a lesser extent D571 are the major sites for initial salt-bridge formation between the gateway and POPC.

When the gateway/annulus complex (Fig. 2C) was rotated 90˚ around its x-axis, residues of the gateway could be easily visualized through the annulus orifice (Fig. 2D). Amino acid residues 73–74, 77, 371, and 375 formed the perimeter of the orifice (Figs. 1D and 2D). When the structure in Fig. 2D was rotated 180° around its y-axis (Fig. 2E) and the gateway was no longer visible (Fig. 2F), the annulus orifice faced the outward-open transmembrane cavity. The amino acid composition of the gateway–annulus orifice complex is illustrated in Fig. 2G.

### Salt-bridge formation by the gateway mediates the extraction of POPC from the membrane mound

These observations raise the possibility that salt-bridge formation in the gateway of ABCA1 helps extract PL molecules from the outer membrane leaflet to create the membrane mound. We tested this hypothesis with CGMD simulations that determined how neutralizing charged residues in the gateway affected the extraction of POPC. For this experiment, we used single and multiple alanine substitutions (K568A and 11CmutA—mutation of all 11 charged residues of the gateway to alanine). The wild type and two mutated ABCA1s were then inserted separately into a membrane bilayer of POPC and subjected to 3 replica sets of CGMD simulation for 10 µs. After 10 µs CGMD simulation, the number of POPC molecules in the lipid mound was reduced about 50% by the K568A mutation and about 70% by the 11CmutA mutation (Supplementary Fig. 2D).

**Fig. 4:**
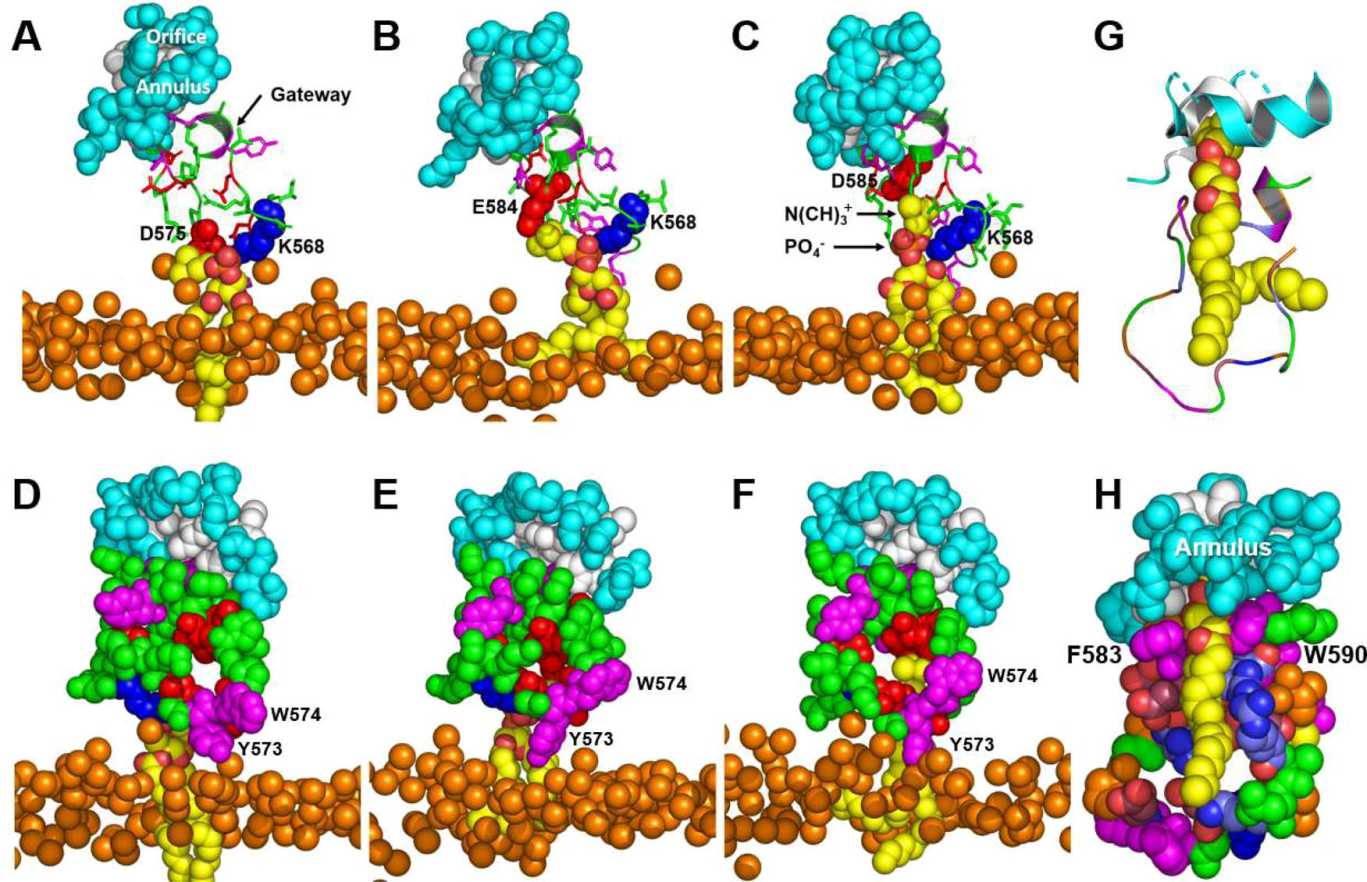
Sequential interactions with gateway residues as an extracted POPC molecule translocates from the external transmembrane cavity to the annulus orifice. **A–C** Salt-bridge formation of acidic amino acid residues D575, E584, and D585 with the N(CH)_3_^+^ headgroup moiety of POPC as the lipid molecule moves from the monolayer toward the annulus during MD simulation. Gateway, green cartoon with attached stick amino acid residues (aromatic residues, magenta); annulus, cyan space-filling; annulus orifice, white space-filling; extracted POPC, yellow space-filling; salt bridged amino acid residues, space-filling; location of outer monolayer, space-filling phosphorus atoms. **D–F** Changes in the conformation of the Y573/W574 aromatic cluster shield the PO_4_^−^ and acyl side chains of POPC during translocation of the lipid molecule from the monolayer toward the annulus during MD simulation. **G**, **H** Van der Waals contacts of POPC upon its approach to the annulus orifice during SMD simulation of the translocation of POPC. **G** Protein in cartoon mode. **H** Protein in space-filling mode. Color code: annulus orifice, white; gateway—aromatic residues, magenta; basic residues, blue; acidic residues, red; hydrophobic residues, orange; other residues, green.

Examination of the frames in the last 5 µs of the three 10 µs CGMD simulations of wild-type ABCA1 revealed that a single POPC molecule was extracted from the outer surface of the plasma membrane (Fig. 4A–C). The PO_4_^−^ group of the POPC formed salt bridges with K568 in 77% of the frames, with R565 in 3% of the frames, with K570 in 7% of the frames, and with R579 in 5% of the frames; no salt bridges were formed with R587 (Supplementary Table 1). During the simulation, POPC progressively moved 10, 12, 15, and 17 Å above the outer monolayer surface as it formed sequential salt bridges with D571, D575, E584 and D585, respectively (Fig. 4A–C). The Y573/W574 cluster at the bottom of the gateway also rearranged to shield the extracted POPC from the outer membrane monolayer as it moved toward the annulus (Fig. 4D–F).

ABCA1 with the K568A mutation also extracted a single POPC molecule from the outer leaflet of the membrane but had a remarkably different pattern of salt bridges from the wild type; the PO_4_^−^ group of the POPC formed salt bridges with R565 in 5% of the frames, with K568 in 0% of the frames, with K570 in 0.2% of the frames, with R579 in 11% of the frames, and with R587 in 0% of the frames (Supplementary Table 1). The 11CmutA mutation exhibited a flat membrane surface with no salt bridges to POPC and no lipid extraction during the entire CGMD simulation (Fig. 3A, right panel).

To determine if charged amino acids in the gateway also help export PL from cells, mutations K568A and 11CmutA were created in constructs of human ABCA1. When wild-type and mutant ABCA1 proteins were expressed in BHK cells, the K568A mutation reduced PL and cholesterol efflux by APOA1 by ~65% and ~60%, respectively (Fig. 3B). The 11CmutA mutation caused ~90%–95% reduction. Similar results were observed with L-4F, an APOA1-mimetic peptide^18^ (Fig. 3B). Further, a control mutation, Y573F, had little effect on cholesterol or PL efflux. It is important to note that none of the mutations affected cell-surface expression of ABCA1 (Fig. 3B).

To test the possibility that the gateway’s charged and aromatic residues help translocate POPC to the annulus orifice, we conducted two additional mutagenic experiments. Mutation of a cluster of three negatively charged residues (D581K/E584K/D585K) to lysines in the gateway near the annulus reduced cholesterol and PL efflux by 80%–85%. The double mutation F583K/W590E in the upper gateway reduced cholesterol efflux by 95% (Fig. 3B). Neither mutation affected cell-surface expression of ABCA1 (Fig. 3B). Thus, the interactions between the charged residues and hydrophobic residues with POPC that we identified in our simulations fit the extracellular phospholipid translocase model well (Fig. 4).

### POPC movement unwinds the annulus

There are potentially three energy barriers to spontaneous movement of POPC through the annulus orifice into the elongated hydrophobic tunnel. The first is the energetic cost of exposing the acyl chains of POPC to solvent as they move up the gateway to the annulus. The second is the movement of the charged headgroup of POPC through the hydrophobic outward-closed transmembrane cavity and into the annulus orifice. The third is the barrier that would be posed by Van der Waals forces if POPC tried to pass through the initially narrow annulus orifice. Overcoming these possible energy barriers would require some form of power stroke, such as closing the outward-open transmembrane cavity.

Using steered molecular dynamics (SMD), we simulated the movement of a POPC molecule from the membrane mound to the annulus and the elongated hydrophobic tunnel (Fig. 1D–F and Supplementary Fig. 1). The PO_4_^-^ moiety of the POPC initially formed a salt bridge with residue K568 of the gateway, while the N(CH_3_)^+^ moiety formed a salt bridge with D571 (Figs. 1D and 2B). The POPC monomer then migrated through the gateway (Figs. 1D–F and 2D–F) to the annulus at the mouth of the elongated hydrophobic tunnel (Supplementary Fig. 1), contacting residues W590 and R587 of the gateway along the way.

We then used SMD to determine how the annulus changed its structure as extracted POPC moved from the outward-open transmembrane canal through the annulus and into the elongated hydrophobic tunnel. A single POPC molecule (Fig. 4G, H) initially interacted with the gateway (Fig. 4A) and was then translocated through the orifice as its N(CH)_3_^+^ headgroup made Van der Waals contact with the annulus. Two points are noteworthy: (i) two aromatic residues, F583 and W590, created a surface for aligning the POPC acyl chains parallel to the two helices of the annulus (residues 69–80 and 367–379); and (ii) charged residues formed salt bridges with each other to shield the POPC acyl chains on two sides (Fig. 4H).

The conformational changes in the annulus orifice seen during SMD simulations are summarized in Supplementary Fig. 3 and Supplementary Movie 1. We used two pulling velocities (fast, 50 Å/ns and slow, 0.5 Å/ns) for the simulations. The force versus time profile for the two pulling velocities are shown in Supplementary Fig. 4. In both cases POPC passage required input of force. In all of the simulations the choline headgroup of POPC made initial Van der Waals contact with the annulus orifice; then its acyl side chains entered the elongated hydrophobic cavity. As the POPC molecule moved through the orifice the helical region near residue I371 in the annulus partially unwound. The unwound segment regained its helical conformation as the POPC moved through the orifice into the elongated hydrophobic tunnel. The same partial uncoiling of the annulus domain was seen transiently in each of the SMD simulations with each pulling velocity. Hydrogen bonds formed between the PO_4_^−^ group and the exposed helical NH backbone groups at the 2.0 and 7.5 ns timeframes (Supplementary Fig. 5).

**Fig. 5:**
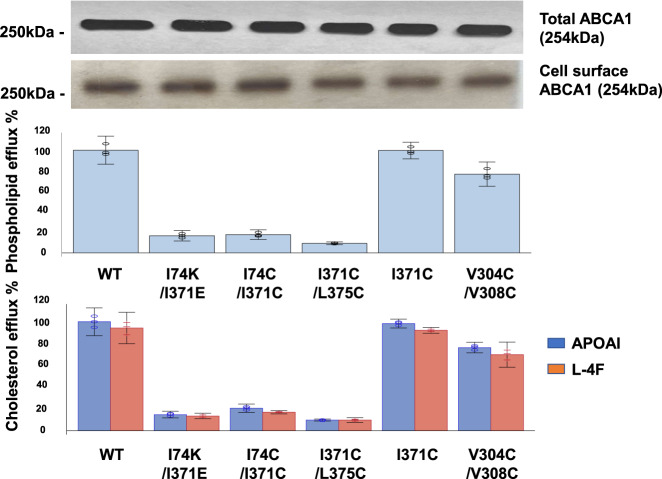
PL and cholesterol efflux by WT ABCA1 and ABCA1 with mutations in the annulus and annulus orifice. WT and mutated ABCA1s (I74K/I371E, I74C/I371C, I371C/L375C, I371C, V304C/V308C) expressed in BHK cells were incubated for 4 h at 37 °C with APOA1 or an equal weight of the APOA1-mimetic peptide, L-4F. PL and cholesterol efflux, and total and cell-surface ABCA1 expression, were determined as described in Methods. Relative cholesterol and phospholipid efflux are normalized to WT and are presented as mean ± SD of three independent experiments with three replicates per experiment. Source data are provided as a Source Data file.

These observations suggest that PL can move from the outward-open transmembrane cavity into the elongated hydrophobic tunnel with conformation changes in the annulus requiring energy input from ATP. To test this hypothesis, we generated three different double mutations in ABCA1 that were predicted to prevent the annulus from opening because of crosslinking by disulfide bond (I371C/L375C, I74C/I371C) or salt-bridge formation (I74K/I371E). We then expressed the wild-type and mutated ABCA1s in BHK cells, and quantified PL and cholesterol efflux. All three double mutations reduced lipid efflux by ABCA1 by 85–90% compared with wild-type ABCA1. Importantly, none of the three mutations affected cell-surface expression (Fig. 5).

Our molecular modeling of ABCA1 suggested that a single I371C mutation or a double i and i + 4 V304C/V308C mutation in the residues adjacent to the annulus orifice would not significantly affect lipid efflux (Fig. 5). When expressed in BHK cells, neither of those mutations in ABCA1 significantly impacted either PL or cholesterol export. Unlike C74–C371 or C371–C375, residue C371, though hydrophobic, is unable to form a disulfide bond to blockade the annulus. C304-C308, which are immediately adjacent to—but not part of—the annular orifice, can form a disulfide bond, but that had little effect on efflux.

### The ATP-free structure of ABCA1 and the homology model of the ATP-bound ABCA1 are respectively in the outward-open and outward-closed transmembrane cavity conformation

Our simulations suggest that ABCA1 transports PL by a mechanism distinct from the alternating-access mechanism and that of other known ABC transporters. To further explore the mechanism of PL export, we constructed a homology model of ATP-bound ABCA1 based on recently published cryo-EM structures of ABCA4^19,20^. We used this approach because the amino acid sequence of ABCA1 is 51% identical and 66% similar to the sequence of ABCA4^21^. Moreover, phylogenetic modeling^22^ shows that the two proteins are among the most closely related ABCA transporters^23^. Finally, it is well established that ABCA4 extracts its substrate from the outer plasma membrane monolayer^19,20^, which is in excellent agreement with our proposed model of ABCA1.

**Fig. 6:**
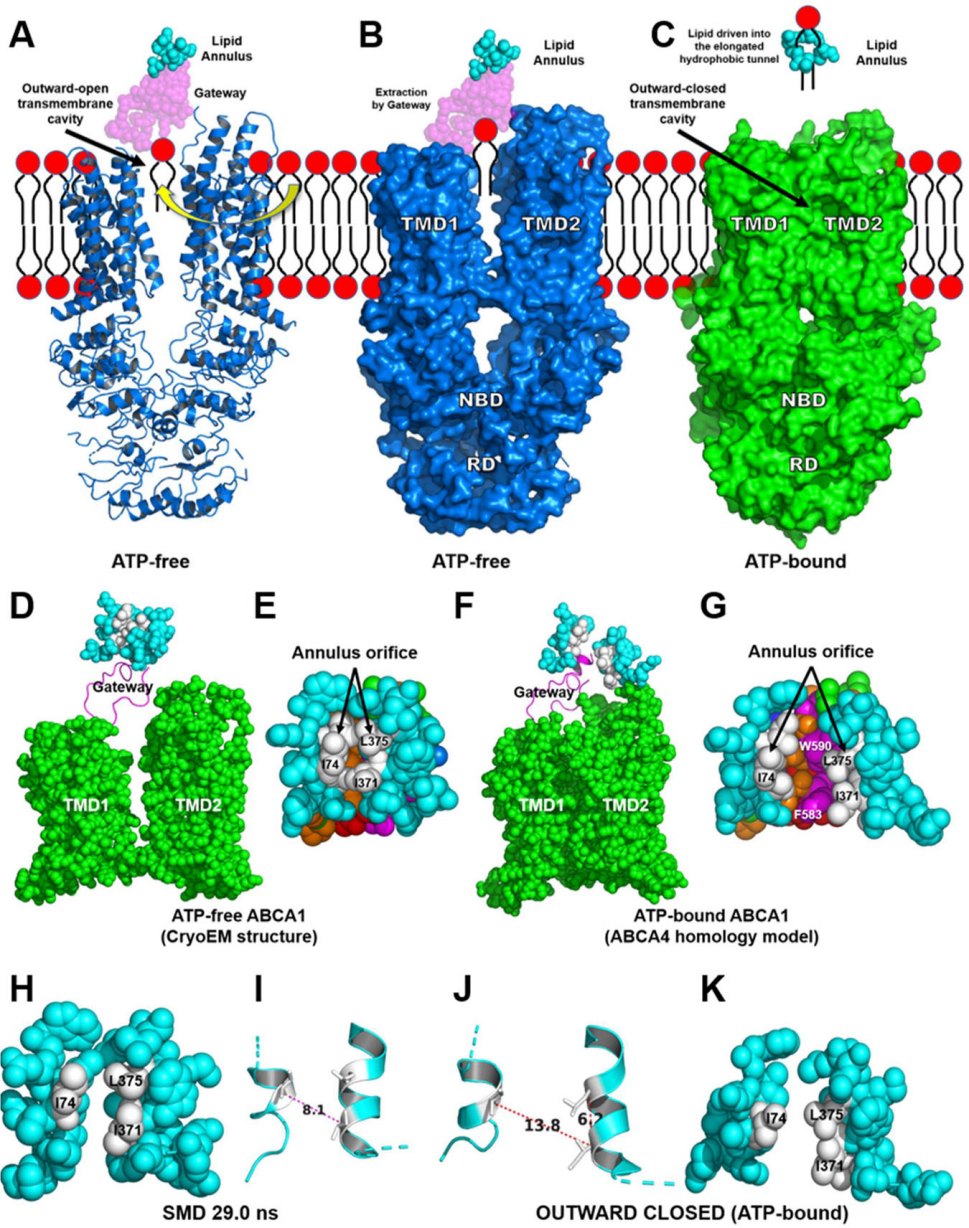
The structures of ATP-free ABCA1 determined by cryo-EM and ATP-bound ABCA1 based on homology modeling with ABCA4. **A** Cartoon of the cryo-EM determined NBDs, cytoplasmic regulatory domains (RDs), TMDs, gateway and lipid annulus of ATP-free ABCA1^13^. POPC has diffused into the outward-facing transmembrane cavity from the outer cell membrane monolayer. **B** Surface representation of the portion of ABCA1 shown in panel A. PC extracted by the gateway is pulled from the outer leaflet of the plasma membrane. **C** Surface representation of ATP-bound ABCA1 derived from a homology model of ATP-bound ABCA4 (the power stroke). The outward-facing cavity of ABCA1 is in the outward-closed conformation. Closure of the cavity drives extracted lipid through the annulus into the elongated hydrophobic tunnel. The annulus orifice opens fully during the power stroke initiated by ATP-binding. **D–G** Structural differences in the annulus and annulus orifice of ATP-free ABCA1 determined by cryo-EM and ATP-bound ABCA based on homology modeling with ABCA4. **D**, **F** The annulus, gateway and TMDs in the ATP-free and ATP-bound forms of ABCA1, respectively. Annulus, space-filling cyan; gateway, magenta cartoon; TMD1-2, space-filling green. **E**, **G** The annulus orifice in the ATP-free and ATP-bound forms of ABCA1, respectively. Note the difference in size of the orifices in the two forms of ABCA1. **H**, **I** Space-filling and cartoon figures, respectively, of the annulus (cyan) and orifice (white) of the 29.0 ns frame of a SMD simulation. Note the 8.1 Å; Cα distance—I74 to I371—between the two helices. **J**, **K** Cartoon and space-filling figures, respectively, of the annulus (cyan) and annulus orifice (white) from the outwardly closed ATP-bound ABCA4-homology model. Note the 13.8 Å Cα distance—I74 to I371—between the two helices.

In the cryo-EM structure of ATP-free ABCA1, the transmembrane cavity is outward-open (Fig. 6A, B). In contrast, our homology model of ATP-bound ABCA1 resembles the cryo-EM structure of ABCA4 because it shows no significant substrate-binding transmembrane cavity on either side of the membrane leaflet (Fig. 6C), a conformation we term outward-closed. This homology model suggests that ATP-binding changes the conformation of ABCA1 while providing the energy for translocating POPC through the annulus orifice and into the elongated hydrophobic tunnel.

We hypothesized that the change from the outward-open to outward-closed conformation of ABCA1 might also open the transporter’s annulus orifice. Examination of the two conformations revealed that the orifice’s two helices moved 6 Å further apart, providing space for one or more POPC molecules to pass through (compare Fig. 6H, I with 6J, K). Our observations suggest that ATP-driven changes in the conformation of ABCA1 from the outward-open to the outward-closed form provides the energy for translocating PL from the outward-facing cavity, into the annulus, through the annulus orifice, and into the elongated hydrophobic tunnel.

As noted by Xie et al.^20^ the outward-open conformation of ABCA4 exhibits a well-ordered S-loop (residues ~337–353) that is located just above the plane formed by the membrane outer leaflet of the outward-open structure. In their nomenclature, S stands for substrate; they show the substrate, N-retinylidene-phosphatidylethanolamine, is coordinated to aromatic residues W339, Y340, Y345, and F348 in a small helical portion of the S-loop. Close inspection reveals that this region is completely disordered in all cryo-EM structures of ABCA1, including ATP-free^13^, such that Qian et al. did not attempt to model that region.

When we aligned the ATP-free ABCA1 structure with the ATP-free ABCA4 structure, the gateway domain in ABCA1 (residues 564–592) aligns with an analogous domain in ABCA4 (residues 579-607) (Supplementary Fig. 6). The most significant compositional difference between the two aligned gateway domains is the addition of an extra basic residue by replacement of G572 in ABCA1 with R587 in ABCA4. We postulate that this additional basic residue is responsible for creation of the S-loop by formation of a salt bridge between the gateway and E341 in the S-loop (Supplementary Fig. 6).

**Fig. 7:**
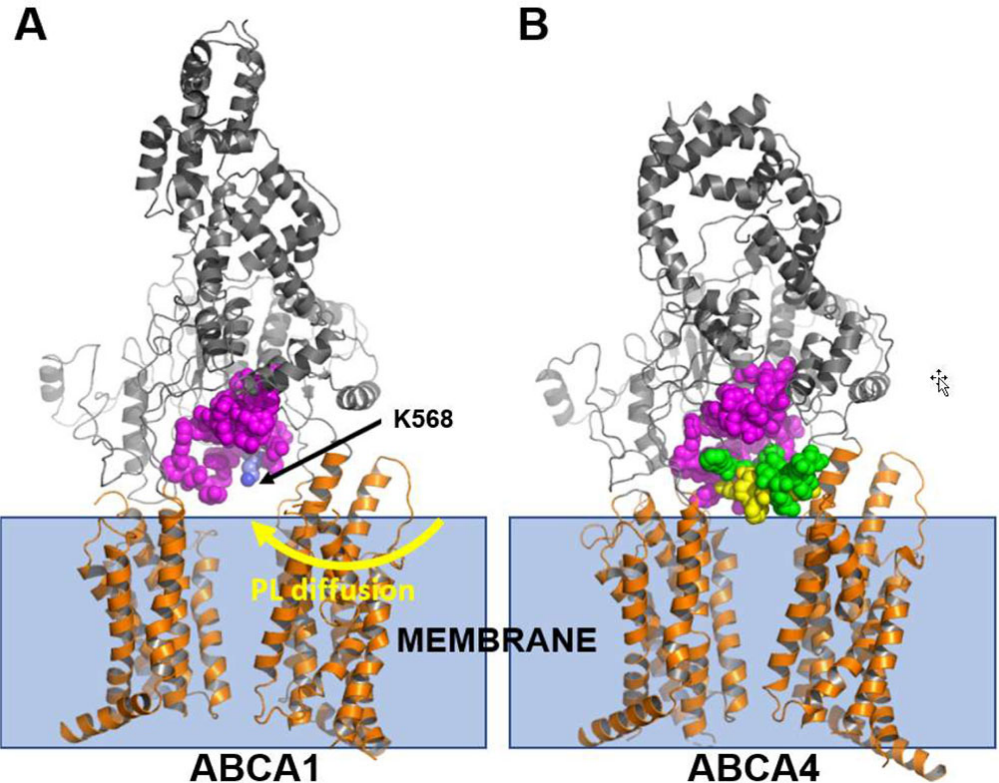
Structural features of ABCA1 and ABCA4 may contribute to their transporting substrates in opposite directions. Our model for substrate transport by ABCA1 proposes that the negatively charged phosphate group of PL in the outer leaflet of the membrane initially interacts with the positively charged residue K568. It then moves through the gateway (**A**, magenta) and into the elongated hydrophobic tunnel. The ATP-free structure of ABCA4 exhibits a structure analogous to the gateway (**B**, magenta). An extended loop, termed the S-loop (green, space-filling) because it binds N-retinylidene-phosphatidylethanolamine (**B**, yellow space-filling), resides adjacent to the gateway of ABCA4. The location of the S-loop in the outward-facing transmembrane cavity of ABCA4 may prevent movement of N-retinylidene-phosphatidylethanolamine into the gateway of ABCA4.

ABCA1 and ABCA4 are known to transport substrate in opposite directions, toward and away from the extracellular space, respectively. Not only does the location of the S-loop in the outward-facing transmembrane cavity block contact of PL with the analogous gateway in ABCA4 upon diffusion from the outer membrane monolayer into the outward-open transmembrane cavity (Fig. 7) but the location provides an interesting additional confirmation of our model. The S-loop/gateway complex forms a salt bridge with K583—the residue analogous to the key PL-extracting residue in ABCA1, K568 (Supplementary Fig. 6). In future studies, it will be important to further clarify the structural features of ABCA1 and ABCA4 that permit them to transport substrates in opposite directions.

**Fig. 8:**
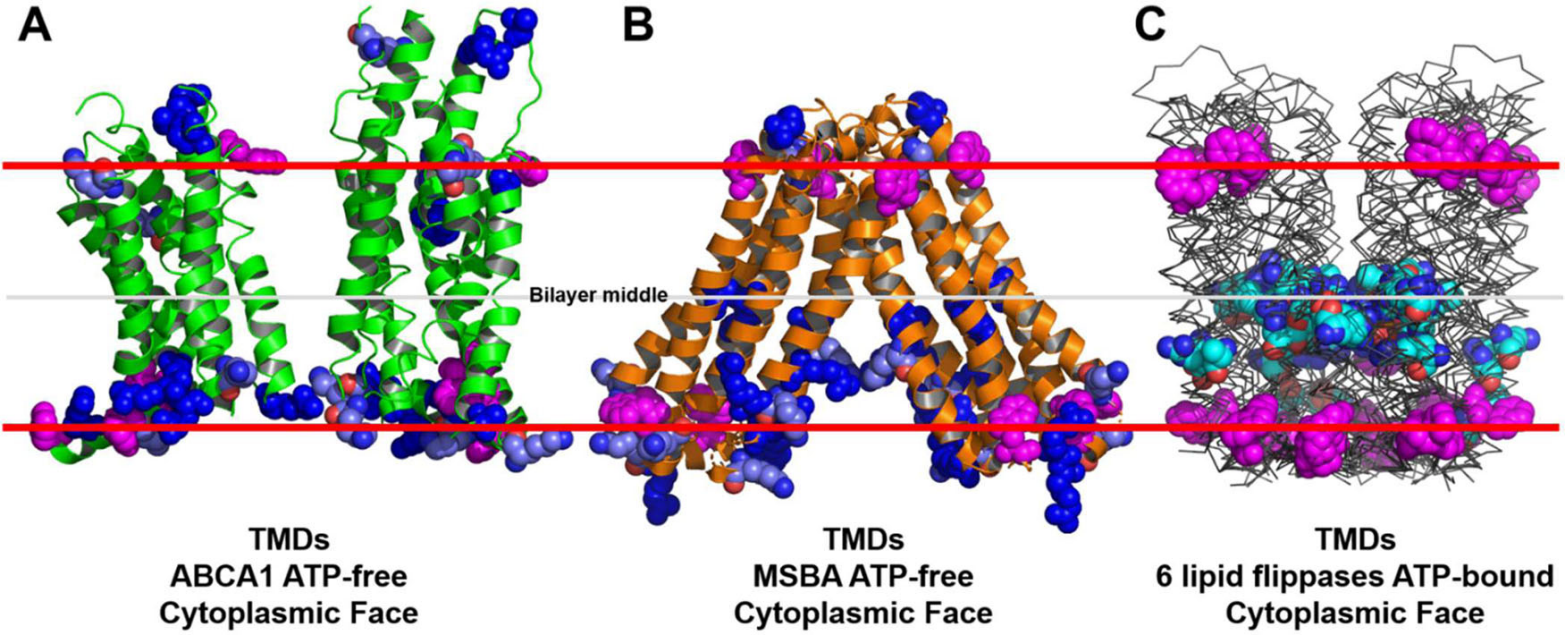
Basic residue distribution in the TMDs of ABCA1 and six established lipid flippases. The locations of tryptophan residues (space-filling magenta) are used to mark the membrane interfaces. In **A**, **B**, lysine residues and arginine residues are pale blue and dark blue, respectively. In **C**, intramembrane lysine residues and arginine residues are cyan. **A** ATP-free human ABCA1 (PDB entry 5XJY)^13^. **B** ATP-free S. Typhimurium MsbA (PDB entry 6BL6)^62^. **C** Six aligned structures of MsbA orthologs from three different bacterial species (PDB entries 2HYD, 3B5Z, 3B60, 5TTP, 5TV4, and 6BPP)^63–66^.

### ABCA1 lacks clusters of charged amino acid residues in the middle of its transmembrane domain (Fig. 8A)

Our findings contrast with the alternating-access mechanism of substrate export used by ABC transporters with floppase activity. Those transporters switch between an open, inward-facing transmembrane cavity and a closed, outward-facing transmembrane cavity^17^. Moreover, most of their structures reveal multiple basic amino acid residues (Fig. 8C) or, in the case of ABCB4^24^, a single histidine residue in the middle of the TMD. In contrast, the cryo-EM structure of ABCA1 shows only hydrophobic amino acids in the middle of the TMD. In the ATP-free conformation, there is only one histidine residue 5 Å or so toward the cytoplasmic side of the membrane and not in the outward-open transmembrane cavity.

## Discussion

Our studies suggest that a key early step in PL export by ABCA1 is extraction of PL molecules from the outer leaflet of the plasma membrane into the transporter’s outward-open transmembrane cavity, which has an orifice into its interior. Homology modeling of ATP-bound ABCA1 using the ABCA4 cryo-EM structure suggests that closure of the outward-open transmembrane cavity drives lipid out of the cavity. Our model thus indicates that ABCA1 acts as an extracellular translocase that pumps PL from the outer leaflet of the plasma membrane through its gateway and into an elongated hydrophobic tunnel. Importantly, this interpretation is consistent with the outward-open transmembrane cavity of nucleotide-free ABCA1 determined by cryo-EM^13^ and of the outward-closed ATP-bound ABCA1 we produced by homology modeling of ABCA4. In striking contrast, the alternating-access model predicts that nucleotide-free ABCA1 should exhibit an inward-facing transmembrane cavity and that ATP-bound ABCA1 should have an outward-facing one^5^.

The robust fit of our mutagenesis studies with the extracellular phospholipid translocase model demonstrates the power of the Martini-based CGMD simulations for the investigation of the function of integral membrane proteins^25–27^. It is important to note that we used a simplified model membrane for our studies, and that pure POPC membranes are only a first approximation to plasma membranes. In future work it will be important to extend our observations using complex mixtures of lipids that more closely mimic the plasma membrane^28^.

Phillips and colleagues have shown that ABCA1 in cultured macrophages promotes the formation of three classes of extracellular lipid particles^29^. In the presence of APOA1, two of the classes (9 nm and 12 nm in diameter) contain APOA1, HDL’s major protein. Although 12 nm HDL accounts for only a very small fraction of circulating HDL in humans, 9 nm particles closely mimic the size of HDL found in LCAT-deficient subjects (8 nm). These small particles are thought to be nascent HDL generated by ABCA1. In Phillips’ study^29^, the 9 nm particles were 66% PC, 9% SM, 7% PE, 5% PS, and 9% PI. It is well established that PC and SM are located predominantly in the extracellular side of the plasma membranes of mammalian cells^28^. The finding that ~75% of the phospholipids in the small HDL particles were outer leaflet lipids is in excellent agreement with our proposed model.

ABCA1 reconstituted into liposomes uses ATP to actively transport PL from the cytosolic to exocytoplasmic leaflets^30^. If the alternating-access mechanism were involved, PL would have to rotate through 180° inside the transporter’s TMD. ABC transporters that use the alternating-access mechanism to flop charged substrates have counter-charged amino acids in the middle of the TMD (Fig. 8). This property is essential for moving charged substrates completely across membrane bilayers because opposing charges on a transporter reduce the energy required to move PL across the bilayer, which would otherwise be very costly^31^. Given the lack of charged groups in the middle of the TMD of monomeric ABCA1, such a rotation would be energetically unfavorable. Collectively, these observations strongly suggest that ABCA1 does not use the alternating-access mechanism to flop PL from the cytoplasmic face to the extracellular face of the membrane but is instead an extracellular PL translocase.

We identified the gateway as a 29-residue charged loop (residues 564–592) of ABCA1 between the outward-open transmembrane cavity and the elongated hydrophobic tunnel^13^. CGMD simulations of human ABCA1 embedded in a POPC bilayer showed that the gateway extracts the membrane mound—a 3–5-molecule cluster of monolayer POPC—by lifting it above the extrafacial surface of the plasma membrane. The amino acid residues lining the elongated hydrophobic tunnel that are in Van der Waals contact with the gateway form the annulus. The orifice of the annulus is situated to accommodate a POPC molecule during translocation into the elongated hydrophobic tunnel. Finally, we show how the presence of the blocking S-loop in ABCA4 reverses the direction of substrate translation from extracellular (ABCA1) to intracellular (ABCA4).

The physiological relevance of our CGMD results is suggested by the deficits in Tangier disease. In this disorder, any one of eight mutations in ECD-1, which forms the large exocytoplasmic domain of ABCA1, can impair cholesterol export from cells. Four of these mutations—D571G, R587W, W590S, and W590L^32^**—**reside in the gateway. D571G would block salt-bridge formation with the PL headgroup and could affect the gateway’s structure. R587W would block handoff salt-bridge formation from D571. W590S would block hydrophobic contact with the acyl chains of PL; both W590S and W590L would block π–π interaction with F583. Of the other four mutations, one is near the gateway and three line the elongated hydrophobic tunnel. Consistent with the hypothesis that the gateway plays an important role in PL export, 25 of its 29 residues are 100% conserved in 34 species of ABCA1, from mammals to bony fish. Of the 53 amino acid residues that make up the gateway/annulus complex, 46 are highly conserved.

Although our gateway–annulus model provides strong evidence that PL is translocated into the elongated hydrophobic tunnel of ABCA1, it does not address how translocation subsequently induces cholesterol efflux and the formation of nascent HDL. One hypothesis is that cholesterol efflux is driven by a concentration gradient: free cholesterol in the plasma membrane migrates into cholesterol-free PC exported by ABCA1^33^. Our demonstration that PC in the elongated tunnel is subsequently exported into the extracellular milieu is consistent with this proposal. It should be noted that our model is also consistent with the proposal that lipid buildup in the monomeric ECD triggers ABCA1 to dimerize and bind to APOA1 to produce nascent HDL^16^. However, our model does not explain how ABCA1 could create an active plasma membrane surface where lipid-free APOA1 could be transformed into a nascent HDL particle^34^. In future studies, it will be important to determine how PL translocation into the elongated hydrophobic tunnel of ABCA1 feeds distal events that promote cholesterol efflux and HDL biogenesis.

Our model of ABCA1 as an extracellular PL translocase suggests a unique transport mechanism that differs substantially from mechanisms described for the other members of the ABC transporter superfamily^13,35^. This surprising finding highlights the remarkable diversity in substrate transport within the ABCA transporter superfamily.

## Methods

### Coarse-grained molecular dynamics of POPC

We embedded ABCA1 in a membrane bilayer of POPC and used CGMD to simulate interactions between the transporter and the lipid^36^. This approach requires ~100 times less time to calculate molecular trajectories than with all-atom calculations^37^. Limitations of this approach include the inability to explicitly model hydrogen bonds and helix-to-coil and coil-to-helix transitions in the protein^36^. To increases the stability of amphipathic protein helical domains during interactions with polar lipids, we used an elastic network to produce coarse-grained descriptions of ABCA1 as a network of coupled harmonic oscillators^38^.

The cryo-EM structure of human ABCA1 (PDB entry: 5XJY)^13^ that we used in our model was taken from the Protein Data Bank (PDB). Missing loops were modeled using loopModel.pl script in MMTSB toolset^39^ and MODELLER v9.19^40^. Next, the protein was embedded in 792 POPC membrane bilayers, using the CHARMM-GUI Martini Bilayer Maker^41^. To ensure the protein was in the proper orientation, we manually oriented it to become orthogonal to the lipid bilayer. The system was then solvated by coarse-grained water molecules and neutralized by adding Na^+^ and Cl^-^ ions with 0.15 M ionic strength, using *gmx solvate* and *gmx genion* modules, respectively.

The system was minimized for 5000 steps, using the steepest descent algorithm, and subjected to 35 ns equilibration. Three replica sets of 10 µs CGMD simulations using different random initial velocities for wild-type, K568A, and 11CmutA ABCA1 were performed, each with a time step of 20 fs, using GROMACS version 5.1^42^ with the MARTINI force field 2.1^43,44^ and the elastic network model^38^. The overall shape of the protein was maintained by using the elastic network model in which harmonic potentials (*k* = 1000 kJ mol^−1^ nm^−2^ for secondary structures, k = 500 kJ mol^−1^ nm^−2^ for the rest) were applied on the backbone beads within 0.5 and 0.9 nm. During CG simulations, constant temperature was maintained at 310 K, using the velocity rescaling thermostat^45^ with a coupling constant of 1 ps. Constant pressure was maintained semi-isotropically at 1 atm, using the Parrinello-Rahman barostat^46^ with a coupling constant of 12 ps and a compressibility of 3 × 10^−4^ bar^−1^. CG trajectories of the system were saved every 5 ns. CG structures of protein and the POPC bilayer were extracted from selected frames and converted to all-atom (AA) structures, using the initram.sh script^47^. All figures were generated using PyMol version 2.5.2^48^.

The same protocols were applied to all CGMD simulations (wild-type, K568A, 11CmutA). To identify the key residues responsible for lipid efflux, we mutated selected residues in the gateway and annulus, using the Mutate Residue plugin of VMD version 1.9.4^49^. The cavities in the extracellular domain were detected and visualized by surface representation in PyMol, with cavities and pocket (culled) and cavity detection radius and cavity detection cutoffs of 8 and 4 solvent radii, respectively.

### Steered molecular dynamics simulation of POPC

Protein and POPC bilayers were extracted from selected frames of the coarse-grained simulation of their wild-types and converted to an all-atom structure. The system was solvated by 190,171 TIP3P water molecules, using VMD solvate plugin^49^. Then 268 Na^+^ and 298 Cl^−^ were added to neutralize the system with 0.15 M ionic strength, using VMD ionize plugin^49^. The system was subjected to energy minimization, using the conjugate gradient algorithm followed by 5 ns equilibration. SMD simulations were performed for 0.79 ns (fast pulling) and 30 ns (slow pulling) using NAMD 2.13^50^ with the CHARMM36 force field^51^.

During the SMD simulation, Cα atoms in secondary structures were restrained with the force constant of 1 kcal/mol Å^2^, and a nitrogen atom in the headgroup of 1 POPC was pulled with two constant pulling velocities (fast, 50 Å/ns and slow, 0.5 Å/ns) and the force constant of 14 kcal/mol Å^2^ in two phases: (i) from the gateway (near D571) to the entrance of annulus and (ii) from the entrance of the annulus to the cavity in the ECD along the z-axis. Temperature and pressure were maintained at 310 K and 1 atm using Langevin thermostat and Langevin Piston^52^, respectively. Trajectories were saved every 10 ps.

### Calculation of the number of POPCs lifted by the gateway in wild-type and mutated ABCA1

We used a total of 3000 (1000 frames per set) frames from the last 5 µs of 10 µs for wild-type, K568A, and 11CmutA (mutation of all 11 charged residues to alanine) for this analysis. First, the center of mass of the phosphorus atoms of the upper POPC bilayers was calculated. Then, the z-distance between a POPC in the tunnel formed by TMD and the center of mass of the phosphorus atoms was calculated. A total of 6 bins was generated based on the distance from 10 to 16 Å, with a bin width of 1 Å, and the number of POPC was counted in each bin.

### Calculation of the number of salt bridges between POPC in the membrane mound and eleven charged residues of the gateway (E564, R565, K568, K570, D571, D575, R579, D581, E584, D585, and R587)

A total of 3000 frames (1000 frames per set) from the last 5 µs of 10 µs for wild-type and K568A were used for the analysis of the CGMD simulations. The cutoff distance for the calculation of the number of salt bridges formed between the choline/phosphate headgroup of POPC and the side chain of charged amino acids was 6 Å.

### Homology model of ABCA1 bound to ATP

Inspection of the atomic models and electron density were performed using the published 2017 structure of ABCA1 (PDB entry 5XJY)^13^, the 2021 structure of ATP-bound ABCA4 (PDB code 7LKZ)^19^, and their corresponding electron density maps (emd_6724 and emd_23410, respectively) obtained from the PDB. Ordered residues in the ABCA4 structure were extracted from PyMol and aligned to ABCA1, using NCBI Cobalt^21^. ABCA1 insertions that did not have counterpart residues in ABCA4 were then removed. Mutagenesis of the ABCA4 model to ABCA1 was then performed with scripts written in Perl and PyMol version 2.0.6^53^, which select the best rotamer side chain confirmations based on least clashing with other atoms in the model. The final model was then energy-minimized using Phenix^54^.

### APOA1-mimetic peptide

The APOA1-peptide mimetic L-4F induces cholesterol efflux from cells overexpressing ABCA1^55^ to a degree rivaling that of APOA1 itself^56,57^. We hypothesized that L-4F would bind directly to extracellular lipid domains formed by ABCA1 independently of APOA1-binding domains and, because of its high lipid affinity compared to APOA1^18^, would effectively solubilize these domains without requiring a specific binding domain. We included L-4F in our lipid efflux assays, using weight-to-weight standardization of APOA1 to L-4F, on the assumption that both APOA1 and 4F efflux lipid by forming discoidal particles with comparable double belt structures^58^.

### Cholesterol and PL efflux

Cells were incubated overnight at 37 °C with 1 μCi/ml of [^3^H]-cholesterol (PerkinElmer Life Sciences) or [^3^H]choline (PerkinElmer Life Sciences), respectively, to quantify cholesterol and PL efflux. Cells were washed once, incubated for 18 h, washed once, and then incubated with medium minus or plus 10 μg/ml APOA1 or 10 μg/ml L-4F for 6 h. Medium and cells were harvested and assayed for [^3^H]. APOA1-mediated and L-4F-mediated lipid efflux were calculated as percentages of total [^3^H] released into the medium after subtracting the values obtained in the absence of APOA1^59,60^. Results were normalized to values for cells expressing wild-type ABCA1.

### Total and cell-surface ABCA1 expression

ABCA1 antibody was diluted at 1;1000. To measure ABCA1 protein levels, cell proteins were solubilized with 5% SDS and fractionated by SDS-PAGE. ABCA1 was quantified by immunoblot analysis^60^. Cell-surface ABCA1 was quantified by treating cells for 30 min at room temperature with 1 mg/ml sulfo-N-hydroxysulfosuccinimide-biotin, isolating ABCA1 by immunoprecipitation and SDS-PAGE, and probing nitrocellulose blots with a streptavidin-HRP ECL assay (BioRad)^59,61^.

### Reporting summary

Further information on research design is available in the Nature Research Reporting Summary linked to this article.

## Supplementary information

Supplementary Information

Description of Additional Supplementary Files

Supplementary Movie 1

Reporting Summary

## Data Availability

MD Simulation trajectories are available at https://figshare.com/articles/dataset/MD_simulation_trajectories_used_in_the_paper_ABCA1_is_an_extracellular_phospholipid_translocase_/18095690. The protein structures used in MD simulations (PDB entry 5XJY), homology modeling (PDB entry 7LKZ), and in Fig. 8B (PDB entry 6BL6) and 8C (PDB entries 2HYD, 3B5Z, 3B60, 5TTP, 5TV4, and 6BPP) are available on the Protein Data Bank. Source data are provided with this paper.

## Source data

Source Data

## Code availability

All custom scripts used in this study are freely available at https://figshare.com/articles/dataset/MD_simulation_trajectories_used_in_the_paper_ABCA1_is_an_extracellular_phospholipid_translocase_/18095690.
