# Supplementary material for: ABCA1 is an extracellular phospholipid translocase
Source: Nat Commun. 2022 Aug 16;13:4812. doi: 10.1038/s41467-022-32437-3 (PMC9381790; doi:10.1038/s41467-022-32437-3)
Supplement: Supplementary file 1 — Supplementary Information [file 41467_2022_32437_MOESM1_ESM.pdf]

SUPPLEMENTARY INFORMATION

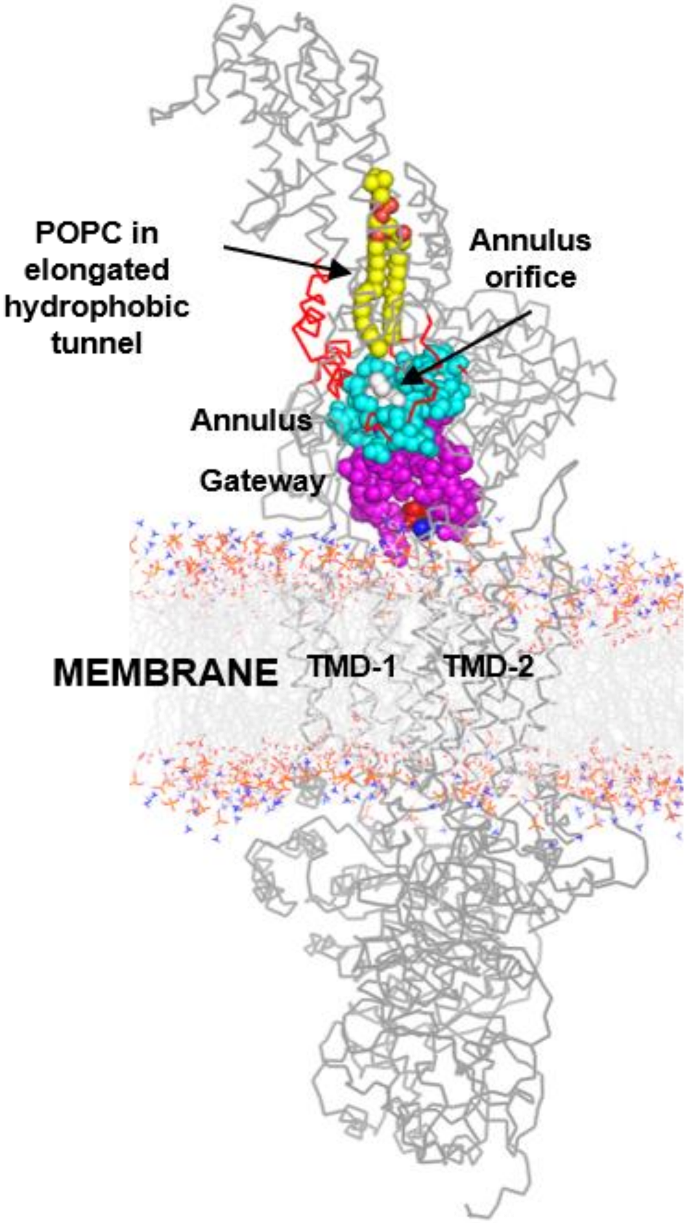

Supplementary Figure 1. Final frame of a SMD simulation of the complete migration of a POPC monomer through the annulus orifice into the elongated hydrophobic tunnel.

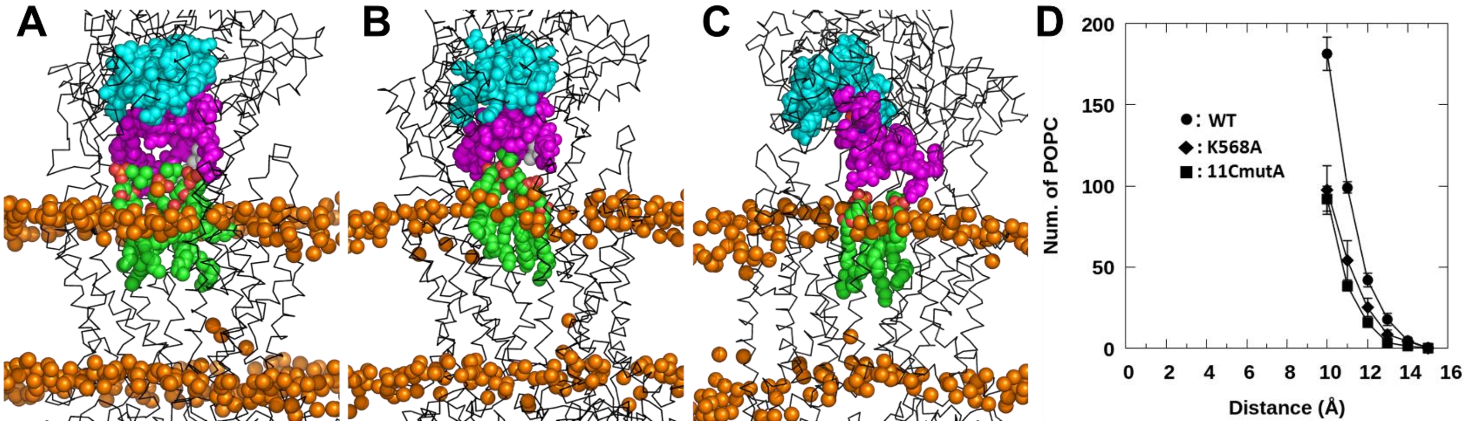

Supplementary Figure 2. The gateway's charged amino acids promote PL extraction, membrane mound formation, and lipid export by ABCA1. All CGMD structures were converted to all-atom models. Extraction of POPC molecules from the membrane bilayer into the gateway by wild-type and mutated ABCA1 **A-C.** Final frames of 2 μsec CGMD simulations showing the gateway (magenta, spacefilling), annulus (cyan, spacefilling) and adjacent bilayer of three different ABCA1 monomers inserted into a POPC bilayer. The monomers were WT, K568A, and 11CmutA (mutation of all 11 charged residues to alanine). Displaced POPC forming the membrane mounds are shown as green, spacefilling molecules. The positions of the bilayer surfaces in each image are denoted by orange space-filling phosphate

A. Wild-type gateway in ABCA1.

B. K568A mutation in the gateway.

C. Mutation of all 11 charged residues to alanines (11CmutA) in the gateway.

D. Effects of gateway mutations on formation of the membrane mound. The plot shows the number of POPC molecules (y-axis) lifted above the outer monolayer surface as a function of how far (x-axis) the P atom of each POPC rises during the last 5 μsec of the 3 replica 10 μsec CGMD simulations of WT, K568A and 11CmutA. Error bars represent the standard deviation from the mean of three simulations. Source data are provided as a Source Data file.

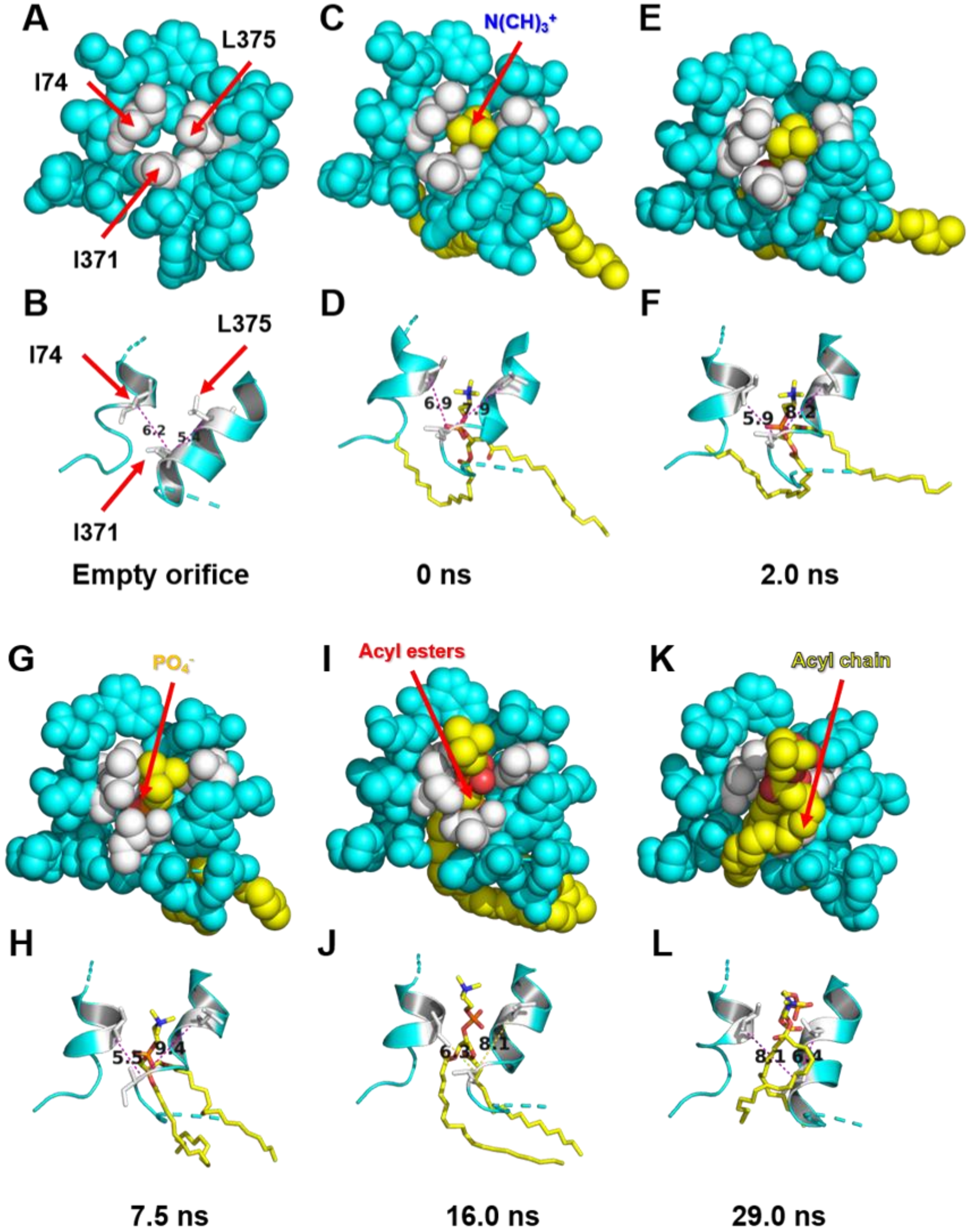

Supplementary Figure 3. Changes in conformation of the annulus orifice during sequential SMD translocation of POPC (slow pulling velocity) from the outward-open transmembrane

cavity to the elongated hydrophobic tunnel.

A-L. Structures of the annulus (cyan) and annulus orifice (white) during POPC (yellow) translocation: A,B) before SMD simulation; C,D) 0 ns ($N(CH)_3^+$ moiety in initial contact with the orifice); E,F) 2.0 ns ($N(CH)_3^+$ moiety moved into the orifice); G,H) 7.5 ns (PO_4^- moiety moved into the orifice); I,J) 16.0 ns (fatty acyl ester moieties moved into the orifice); K,L) 29.0 ns (one fatty acyl chains has just popped out of the orifice). The upper images are spacefilling and the lower images are cartoon. The cartoon images D-J show partial helical unwinding predominately in the region near residue I371 as the POPC headgroup moves through the orifice. The cartoon image L shows the unwinding collapsing back to helix conformation as the headgroup moieties move out of the orifice and into the elongated hydrophobic tunnel. We saw the same transient partial helical unwinding in the same annulus domain in 2 separate SMD simulations using two different pulling velocities. The Cα distances between orifice residues I74 to L375 and I371 to L375 are indicated by magenta dotted lines in the cartoon images; the maximum Cα distances are 8.1 Å between I74 and I371 in panel L (acyl chains) and 9.4 Å between I371 and L375 in panel H. Hydrogen bonds formed by the $N(CH)_3^+$ and PO_4^- headgroup moieties (predominantly the PO_4^- group) with the exposed helical -NH backbone groups in panels F, H are shown in **Supplementary Fig. 5**.

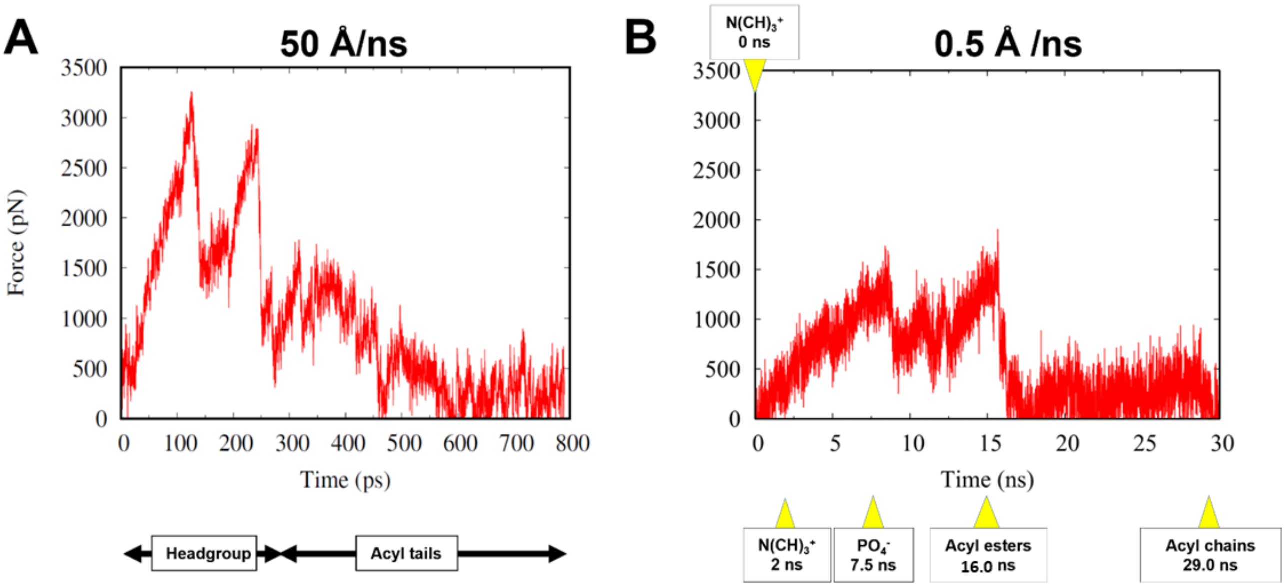

Supplementary Figure 4. Plots of force versus time during fast versus slow SMD translocation of a POPC molecule from initial contact with the annulus orifice to where one of the two acyl chains has moved through the orifice and into the elongated hydrophobic tunnel. The five yellow arrowheads in panel B indicate frames (shown in **Supplementary Fig. 5**) in which the $N(CH)_3^+$, PO_4^-, acyl chain ester headgroup moieties and the fatty acyl chains, respectively, are interacting with the orifice. Plots were generated using gnuplot version 5.2 (http://gnuplot.info). Source data are provided as a Source Data file.

A. Pulling force (fast, 50.0 Å/ns) versus SMD simulation time.

B. Pulling force (slow, 0.5 Å/ns) versus SMD simulation time.

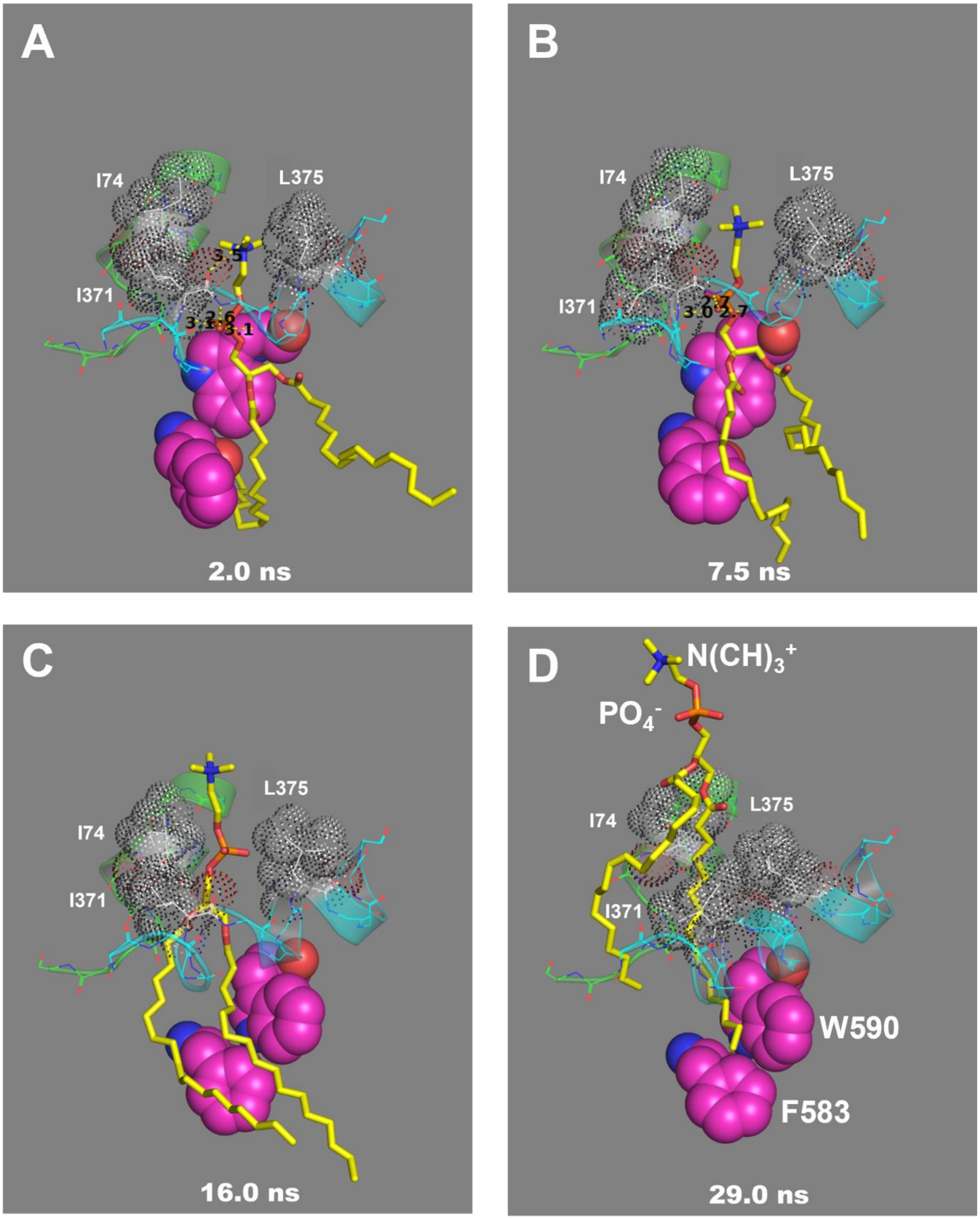

Supplementary Figure 5. Detailed stick images of the POPC headgroup moieties and the two annulus helices (res. 69-80, green; res. 368-379, cyan) as the POPC passes through the annulus at the 2.0 ns **(A)**, 7.5 ns **(B),** 16.0 ns **(C)**, and 29.0 ns **(D)** frames of the SMD shown in

Supplementary Fig. 3. The yellow dotted lines show H-bonds formed by the PO_4^- and $N(CH)_3^+$ headgroup moieties with the partially unraveled helix in panels A and B. Note the previously helical domain opens slightly between panels A and B and closes slightly between panels B and C.

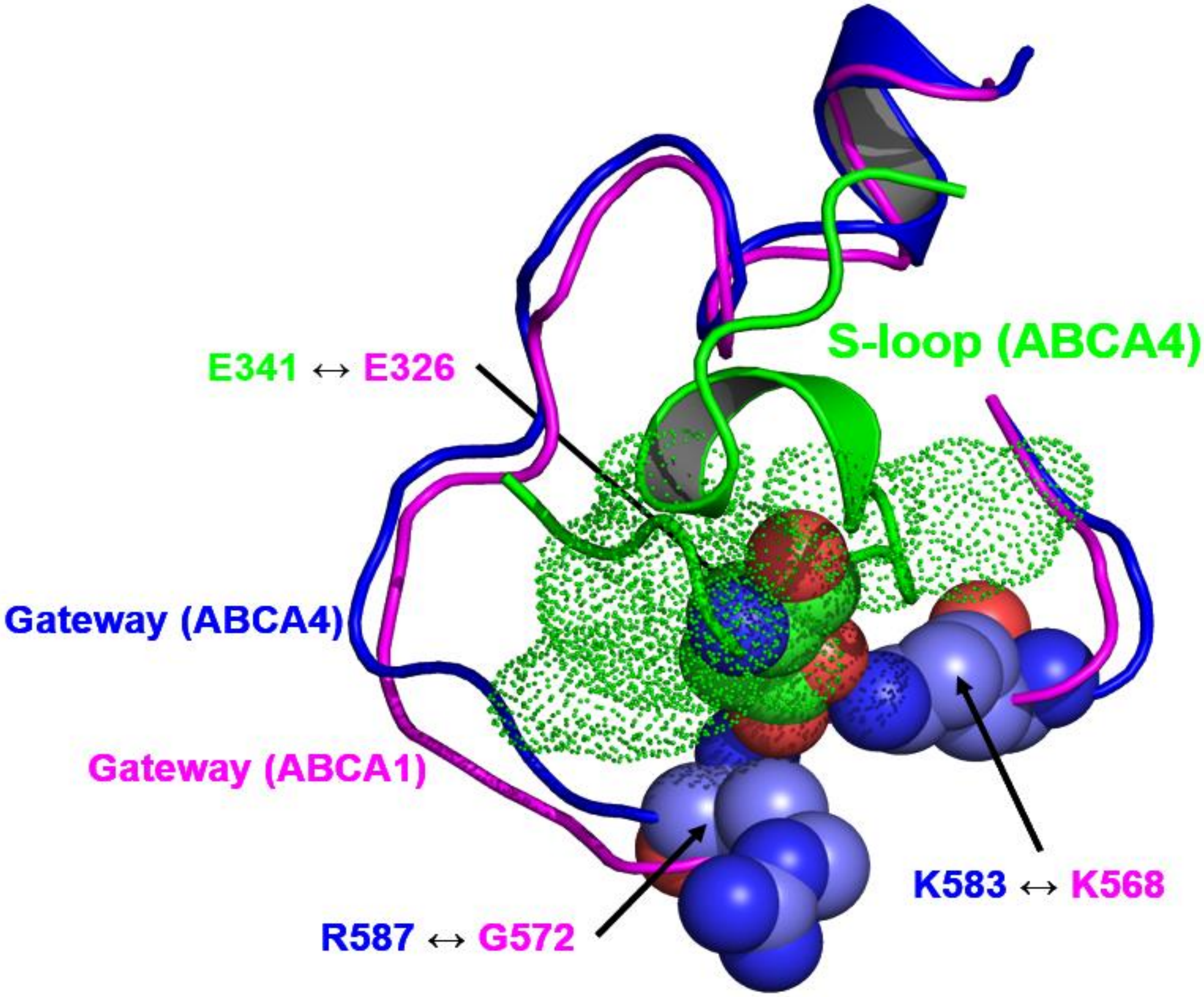

Supplementary Figure 6. Examination of the molecular mechanisms responsible for substrate movement in opposite directions between ABCA1 and ABCA4. Sequence alignment of the ATP-free ABCA1 structure with the ATP-free ABCA4. The image highlights the aligned structures of the analogous gateway regions of the ATP-free forms of the two transmembrane proteins. ABCA1 gateway: residues 564-592, magenta cartoon. ABCA4 gateway: residues 579-607, blue cartoon; residues R587 (analogous to G572 in ABCA1) and K583 (analogous to K568 in ABCA1), blue spacefilling. S-loop in ATP-free ABCA4: residues

337-353, green cartoon; residue E341 (analogous to E326 in ABCA1), green spacefilling; aromatic residues 339, 340, 345, and 348, green dots.

WT SALT BRIDGES		
POPC group	Residue	% of frames
PO_4^-	R565	2.5 ± 1.2
	K568	76.5 ± 2.7
	K570	6.8 ± 1.5
	R579	4.9 ± 0.2
	R587	0.0 ± 0.0
$N(CH)_3^+$	E564	4.9 ± 0.0
	D571	38.9 ± 3.1
	D575	24.4 ± 2.5
	D581	0.0 ± 0.0
	E584	22.7 ± 3.6
	D585	10.5 ± 1.3

K568A SALT BRIDGES		
POPC group	Residue	% of frames
PO_4^-	R565	4.8 ± 1.1
	K568	–
	K570	0.2 ± 0.0
	R579	10.8 ± 2.8
	R587	0.0 ± 0.0
$N(CH)_3^+$	E564	4.1 ± 0.6
	D571	63.5 ± 0.7
	D575	27.1 ± 4.4
	D581	8.2 ± 1.2
	E584	20.3 ± 2.6
	D585	4.8 ± 0.7

Supplementary Table 1. Analysis of the frequency of salt-bridge formation between the extracted POPC molecule and the charged residues of the gateway during the last 5 µsec of the 3 replica 10 µsec CGMD simulations of ABCA1 WT and K568A in a POPC bilayer. The mutation, 11CmutA, of course, formed no salt bridges. Error bars represent the standard deviation from the mean of three simulations. Source data are provided as a Source Data file.
