# Supplementary material for: ABCA1 is an extracellular phospholipid translocase
Source: Nat Commun. 2022 Aug 16;13:4812. doi: 10.1038/s41467-022-32437-3 (PMC9381790; doi:10.1038/s41467-022-32437-3)
Supplement: Supplementary file 2 — Description of Additional Supplementary Files [file 41467_2022_32437_MOESM2_ESM.pdf]

**Description of Additional Supplementary Files**

**File name: Supplementary Movie 1**

**Description: Movie of SMD pulling of POPC molecule from the entrance into the annulus orifice (0 ns) until passage of the first of the two fatty acyl chains into the elongated hydrophobic tunnel (30 ns).** The major annulus orifice residues (I74, I371 and L375) are in gray stick representation. The two upper gateway fatty acyl chain guide aromatics (F583, W590) are in magenta spacefilling. Watch—as the POPC is translocated upward into the elongated hydrophobic cavity—formation of H-bonds between the POPC headgroup domain and partially unwound helix backbone residues near and either side of I371.
