# Supplementary material for: ABCA1 is an extracellular phospholipid translocase
Source: Nat Commun. 2022 Aug 16;13:4812. doi: 10.1038/s41467-022-32437-3 (PMC9381790; doi:10.1038/s41467-022-32437-3)
Supplement: Supplementary file 4 — Reporting Summary [file 41467_2022_32437_MOESM4_ESM.pdf]

nature portfolio

Corresponding author(s): Jere P. Segrest

Last updated by author(s): Jul 12, 2022

Reporting Summary

Nature Portfolio wishes to improve the reproducibility of the work that we publish. This form provides structure for consistency and transparency in reporting. For further information on Nature Portfolio policies, see our Editorial Policies and the Editorial Policy Checklist.

Statistics

For all statistical analyses, confirm that the following items are present in the figure legend, table legend, main text, or Methods section.

n/a	Confirmed	
☐	☒	The exact sample size (*n*) for each experimental group/condition, given as a discrete number and unit of measurement
☐	☒	A statement on whether measurements were taken from distinct samples or whether the same sample was measured repeatedly
☒	☐	The statistical test(s) used AND whether they are one- or two-sided<br>*Only common tests should be described solely by name; describe more complex techniques in the Methods section.*
☒	☐	A description of all covariates tested
☒	☐	A description of any assumptions or corrections, such as tests of normality and adjustment for multiple comparisons
☒	☐	A full description of the statistical parameters including central tendency (e.g. means) or other basic estimates (e.g. regression coefficient) AND variation (e.g. standard deviation) or associated estimates of uncertainty (e.g. confidence intervals)
☒	☐	For null hypothesis testing, the test statistic (e.g. *F*, *t*, *r*) with confidence intervals, effect sizes, degrees of freedom and *P* value noted<br>*Give P values as exact values whenever suitable.*
☒	☐	For Bayesian analysis, information on the choice of priors and Markov chain Monte Carlo settings
☒	☐	For hierarchical and complex designs, identification of the appropriate level for tests and full reporting of outcomes
☒	☐	Estimates of effect sizes (e.g. Cohen's *d*, Pearson's *r*), indicating how they were calculated

Our web collection on statistics for biologists contains articles on many of the points above.

Software and code

Policy information about availability of computer code

Data collection	All-atom and coarse-grained molecular dynamic simulation data were generating using NAMD (version 2.13) and Gromacs (version 5.1) software, respectively.
Data analysis	Missing loops of ABCA1 were modeled using loopModel.pl script in MMTSB toolset and MODELLER v9.19. The protein was embedded in 792 POPC membrane bilayers, using the CHARMM-GUI Martini Bilayer Maker. Selected residues in the gateway and annulus were mutated using the Mutate Residue plugin of VMD version 1.9.4. The number of POPC molecules lifted above the outer monolayer surface vs the distance was calculated using custom Tcl script. The frequency of salt-bridge formation between the POPC and the four charged residues of the gateway was calculated using custom Tcl script. All figures were generated using PyMol version 2.5.2, and the plots in Supplementary Figures were generated using gnuplot version 5.2 (http://gnuplot.info).<br>Homology model of ABCA1 bound to ATP: Ordered residues in the ABCA4 structure were extracted from PyMol and aligned to ABCA1, using NCBI Cobalt. The final model was then energy-minimized using Phenix.<br>All custom scripts used in this study are freely available at https://figshare.com/articles/dataset/MD_simulation_trajectories_used_in_the_paper_ABCA1_is_an_extracellular_phospholipid_translocase_/18095690

For manuscripts utilizing custom algorithms or software that are central to the research but not yet described in published literature, software must be made available to editors and reviewers. We strongly encourage code deposition in a community repository (e.g. GitHub). See the Nature Portfolio guidelines for submitting code & software for further information.

Data

Policy information about availability of data

All manuscripts must include a data availability statement. This statement should provide the following information, where applicable:

- Accession codes, unique identifiers, or web links for publicly available datasets
- A description of any restrictions on data availability
- For clinical datasets or third party data, please ensure that the statement adheres to our policy

MD Simulation trajectories are available at https://figshare.com/articles/dataset/MD_simulation_trajectories_used_in_the_paper_ABCA1_is_an_extracellular_phospholipid_translocase_/18095690. The protein structures used in MD simulations (PDB entry 5XJY), or homology modeling (PDB entry 7LKZ), and in Figs 8B (PDB entry 6BL6) and 8C (PDB entries 2HYD, 3B5Z, 3B60, 5TTP, 5TV4, and 6BPP) are available on the Protein Data Bank. Source data are provided with this paper.

Field-specific reporting

Please select the one below that is the best fit for your research. If you are not sure, read the appropriate sections before making your selection.

☒ Life sciences ☐ Behavioural & social sciences ☐ Ecological, evolutionary & environmental sciences

For a reference copy of the document with all sections, see nature.com/documents/nr-reporting-summary-flat.pdf

Life sciences study design

All studies must disclose on these points even when the disclosure is negative.

Sample size	*Describe how sample size was determined, detailing any statistical methods used to predetermine sample size OR if no sample-size calculation was performed, describe how sample sizes were chosen and provide a rationale for why these sample sizes are sufficient.*
Data exclusions	*Describe any data exclusions. If no data were excluded from the analyses, state so OR if data were excluded, describe the exclusions and the rationale behind them, indicating whether exclusion criteria were pre-established.*
Replication	*Describe the measures taken to verify the reproducibility of the experimental findings. If all attempts at replication were successful, confirm this OR if there are any findings that were not replicated or cannot be reproduced, note this and describe why.*
Randomization	*Describe how samples/organisms/participants were allocated into experimental groups. If allocation was not random, describe how covariates were controlled OR if this is not relevant to your study, explain why.*
Blinding	*Describe whether the investigators were blinded to group allocation during data collection and/or analysis. If blinding was not possible, describe why OR explain why blinding was not relevant to your study.*

Reporting for specific materials, systems and methods

We require information from authors about some types of materials, experimental systems and methods used in many studies. Here, indicate whether each material, system or method listed is relevant to your study. If you are not sure if a list item applies to your research, read the appropriate section before selecting a response.

Materials & experimental systems

n/a	Involved in the study	
☐	☒	Antibodies
☐	☒	Eukaryotic cell lines
☒	☐	Palaeontology and archaeology
☒	☐	Animals and other organisms
☒	☐	Human research participants
☒	☐	Clinical data
☒	☐	Dual use research of concern

Methods

n/a	Involved in the study	
☒	☐	ChIP-seq
☒	☐	Flow cytometry
☒	☐	MRI-based neuroimaging

Antibodies

Antibodies used	Anti-ABCA1 antiboby was from Novus Biological (NB400-164) and was diluted at 1 to 1000.
Validation	The anti-ABCA1 antibody has been validated by Novus biological and all the validation information is available online at Novusbio.com.

Eukaryotic cell lines

Policy information about cell lines

Cell line source(s)	BHK cells were originally from ATCC. All transfected cell lines were verified and available for anyone who requested. No mycoplasma contamination.
Authentication	All cell lines were created double blinded and were authenticated by measuring ABCA1 expression.
Mycoplasma contamination	All cell lines tested negative for mycoplasma contamination
Commonly misidentified lines (See ICLAC register)	No commonly misidentified cell lines.

March 2021
